# Evolutionary Optimization of Protein Folding

**DOI:** 10.1371/journal.pcbi.1002861

**Published:** 2013-01-17

**Authors:** Cédric Debès, Minglei Wang, Gustavo Caetano-Anollés, Frauke Gräter

**Affiliations:** 1 Heidelberg Institute for Theoretical Studies, Heidelberg, Germany; 2 Evolutionary Bioinformatics Laboratory, Department of Crop Sciences, University of Illinois, Urbana, Illinois, United States of America; 3 CAS-MPG Partner Institute and Key Laboratory for Computational Biology, Shanghai, China; National Cancer Institute, United States of America and Tel Aviv University, Israel

## Abstract

Nature has shaped the make up of proteins since their appearance, 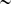
3.8 billion years ago. However, the fundamental drivers of structural change responsible for the extraordinary diversity of proteins have yet to be elucidated. Here we explore if protein evolution affects folding speed. We estimated folding times for the present-day catalog of protein domains directly from their size-modified contact order. These values were mapped onto an evolutionary timeline of domain appearance derived from a phylogenomic analysis of protein domains in 989 fully-sequenced genomes. Our results show a clear overall increase of folding speed during evolution, with known ultra-fast downhill folders appearing rather late in the timeline. Remarkably, folding optimization depends on secondary structure. While alpha-folds showed a tendency to fold faster throughout evolution, beta-folds exhibited a trend of folding time increase during the last 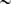
1.5 billion years that began during the “big bang” of domain combinations. As a consequence, these domain structures are on average slow folders today. Our results suggest that fast and efficient folding of domains shaped the universe of protein structure. This finding supports the hypothesis that optimization of the kinetic and thermodynamic accessibility of the native fold reduces protein aggregation propensities that hamper cellular functions.

## Author Summary

Nature has come up with an enormous variety of protein three-dimensional structures, each of which is thought to be optimized for its specific function. A fundamental biological endeavor is to uncover the driving evolutionary forces for discovering and optimizing new folds. A long-standing hypothesis is that fold evolution obeys constraints to properly fold into native structure. We here test this hypothesis by analyzing trends of proteins to fold fast during evolution. Using phylogenomic and structural analyses, we observe an overall decrease in folding times between 3.8 and 1.5 billion years ago, which can be interpreted as an evolutionary optimization for rapid folding. This trend towards fast folding probably resulted in manifold advantages, including high protein accessibility for the cell and a reduction of protein aggregation during misfolding.

## Introduction

The catalog of naturally occurring protein structures [1] exhibits a large disparity of folding times (from microseconds [2], to hours [3]). This disparity is the result of roughly 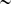
3.8 billion years of evolution during which new protein structures were created and optimized. The evolutionary processes driving the discovery and optimization of protein topologies is complex and remains to be fully understood. Nature probably uncovers new topologies in order to fulfill new functions, and optimizes existing topologies to increase their performance. Various physical and chemical requirements, from foldability to structural stability, are likely to be additional players shaping protein structure evolution. One indicator for foldability, i.e. the ease of taking up the native protein fold, is a short folding time.

Here we propose that foldability is a constraint that crucially contributes to evolutionary history. Optimization of foldability during evolution could explain the existence of a folding funnel [4], [5], into which a defined set of folding pathways lead to the native state, as postulated early on by Levinthal [6]. While the biological relevance of efficient folding still needs to be explored, an obvious advantage is the increase of protein availability to the cell. For instance, folding could decrease the time between an external stimulus and the organismal response. However, this increase of accessibility is probably limited by other factors such as protein synthesis, proline isomerization and disulfide formation. A probably more important point to support folding speed as an evolutionary constraint is that fast folding avoids proteins aggregation in the cell [7]. Aggregation avoidance could lead to a selection of topologically simple structures that fold rapidly or exclusion of a large number of geometrically feasible structures that compromise accessiblity. This could have reduced the catalog of naturally occurring folds [8]–[10].

The balance between the need for new structural designs and functions in evolution and the physical requirements imposing pressure on folding has remained elusive. The increasing number of organisms with completely sequenced genomes and experimentally acquired models of protein structures, combined with new techniques to study the folding behavior of proteins now open new avenues of inquiry. A common approach for such studies has been the use of molecular simulations such as lattice or coarse-grained techniques, which are efficient enough to scan sequence space. Simulations generally involve an algorithm that mimics the evolutionary accumulation of mutations. This allows to monitor how proteins are selected and evolve towards specific features that are optimized, including those linked to folding, structure and function [11]–[13]. In contrast, we have uncovered phylogenetic signal in the genomic abundance of protein sequences that match known protein structures. Specifically, phylogenomic trees that describe the history of the protein world are built from a genomic census of known protein domains defined by the Structural Classification of Proteins (SCOP) [14] and used to build timelines of domain appearance [15], [16] that obey a molecular clock [17]. This information revealed for example the early history of proteins [18], planet oxygenation [17], and the dynamics of domain organization in proteins [19]. All-atom simulations of denatured proteins folding into their native state [20], [21] are computationally too demanding to systematically evaluate the folding times of the available structural models of protein domains, currently 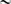
100,000 in total. A decade ago, Baker and co-workers [22] introduced the concept of contact order, a measure of the non-locality of intermolecular contacts in proteins. Contact order was found to be in good correlation with folding times of two state folders but not multistate proteins. Subsequent studies with extended comparison to experiments led to the definition of the Size-Modified Contact Order (SMCO),
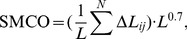
(1)where 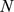 is the number of contacts, 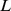 is the total number of aminoacids, and 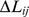 is the number of aminoacids along the chain between residues 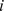 and 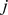 forming a native contact. By correcting for protein size 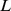, the SMCO showed an improved correlation with experimental folding times, with a correlation coefficient of 0.74 [23].

**Figure 1:**
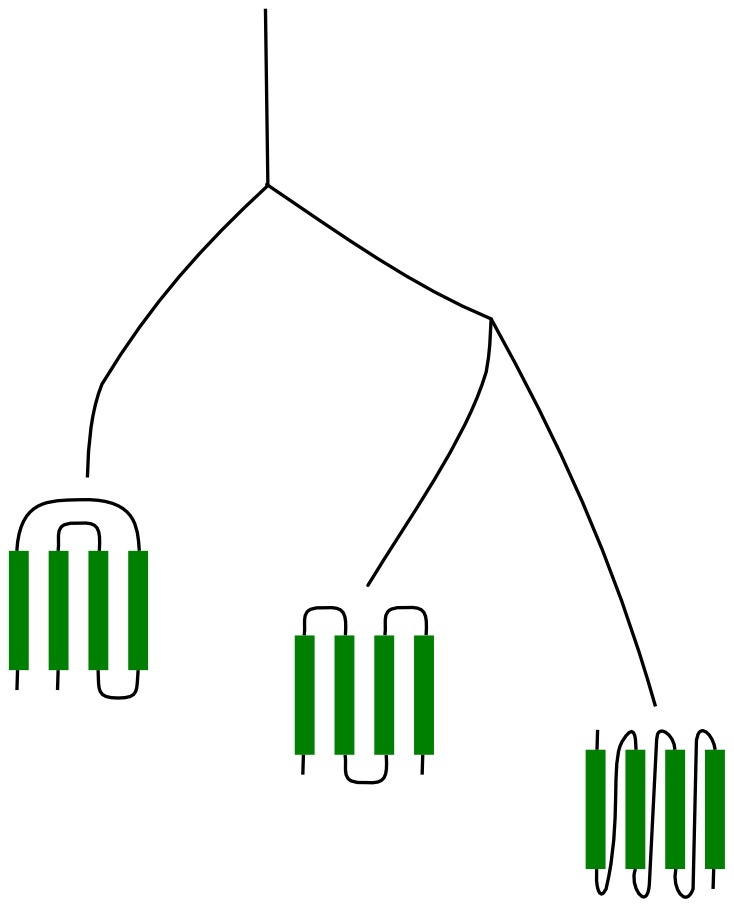
Protein topologies that favor short range inter-aminoacid contacts might be the result of an evolutionary optimization of foldability and thus would have likely appeared late in evolution.

Here, we reveal evolutionary patterns of foldability by mapping the SMCO and thus the folding time onto timelines derived from phylogenomic trees of domain structures (Figure 1). Remarkably, we find there is selection pressure to improve overall foldability, i.e reduce folding times, during protein history. Interestingly, different topologies such as all-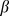 and all-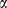 folds show distinct patterns, suggesting folding impacts the evolution of some classes of protein structures more than others.

## Results

### Change in foldability during evolution

**Figure 2:**
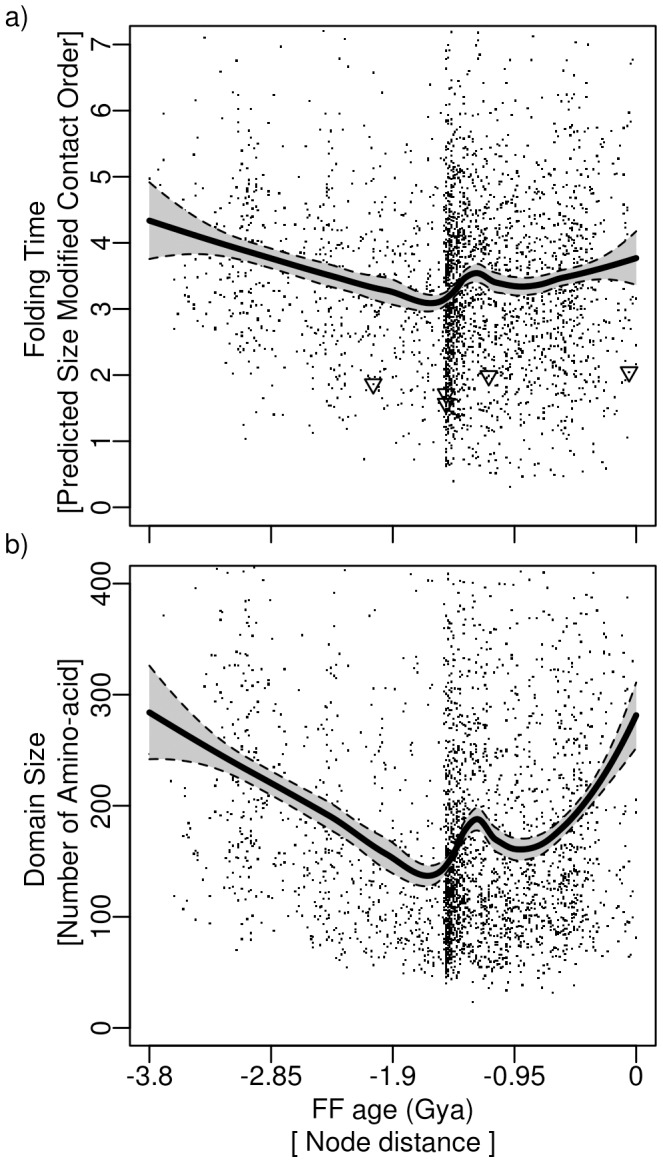
Change in length and foldability during evolution: a) Size Modified Contact Order (SMCO) versus approximative F domain age in billion of years (Gya). Each data point represents an SMCO average of domain belonging to the same F. Triangles show SMCO averages for domains belonging to the same F and experimentally known to be ultra-fast folders [27]. b) Average amino-acid chain length for domains belonging to the same F versus F domain age in Gya. The solid line shows a LOESS polynomial regression, and the grey shade the 95% confidence interval.

To trace protein folding in evolution, we determined the SMCO of protein domain structures at the Family (F) level of structural organization. Figure 2a shows the folding rate of each F, as measured by its average SMCO, as a function of evolutionary time. Using polynomial regression, we observed a significant decrease (p-value = 9.5e-15) in SMCO in proteins appearing between 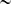
3.8 and 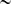
1.5 billion years ago (Gya). Trends were maintained when excluding domains from the analysis solved in multi-domain proteins (Figure S11), and also when studying domain evolution at more or less conserved levels of structural abstraction of the SCOP hierarchy. Namely, we find a significant decrease of SMCO at the level of Superfamily (SF), p-value = 2.6e-15), and at the level of domains with less than 95% sequence identity (p-value< = 2.0e-16, Figure S1a,b). Similarly, consistent results were obtained at the F level using linear regression (p-value = 1.0e-06, Figure S1c). Remarkably, even within a smaller data set of only 87 proteins for which folding times have been measured [24], we find that the experimental folding times exhibit a tendency to decrease early in protein evolution (Figure S2). As an additional way of validation, we repeated the analysis for 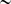
3 million single domain sequences with predicted SMCO [25], and obtained a decrease again of SMCO up to 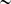
1.5 Gya (p-value< = 2.0e-16, Figures S3, S4). Thus, in this initial evolutionary period, proteins tended to fold faster on average.

As suggested by the decrease in SMCO, during evolution, domains diminish long-range and favor short-range interactions, thereby becoming more strongly connected locally. This picture was further corroborated by an analogous analysis of evolutionary trend in tightness, measured by shortest paths in the network of protein contacts [26]. Tightness, and thus the lengths of paths in the interaction network, decreased in evolution until 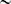
1.5 Gya, followed by an increase, just like the SMCO (Figure S5). Our results support the hypothesis that folding speed acts as an evolutionary constraint in protein structural evolution.

In contrast, we observed an increase in SMCO between 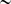
1.5 Gya and the present (Figure 2a). Thus, the appearance of many new stuctures by domain rearrangement 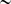
1.5 Gya, also refered to as the “big bang” [19] of the protein world, affected the evolutionary optimization of protein folding. While a linear regression supports the SMCO increase (p-value = 2e-16), it was not as observed at the SF level or at the level of domains (Figure S1a,b), and for the analysis of experimentally determined rates (Figure S2).

Given the observed overall evolutionary speed-up of protein folding, we would expect a late evolutionary appearance of so-called downhill proteins, which feature ultra-short folding times on the microsecond scale. We annotated 11 downhill folders [27] by their Fs, namely a.35.1.2, a.4.1.1, a.8.1.2, b.72.1.1, and d.100.1.1, and show their average SMCO per family as black triangles in the timeline of Figure 2a. All of them, unsurprisingly, have an SMCO 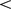
2, and thus fold significantly faster on average than other structures. We find 7% of families to have a lower SMCO (SMCO 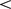
1.5) than the experimentally identified downhill folders. We predict these Fs will fold even faster than the known downhill folders, rendering them interesting candidates for folding assays. The five Fs containing the fast folders have all appeared no earlier than 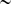
2.5 Gya, suggesting that they are a result of lengthy evolutionary optimization. According to our predictions, the first fast-folding proteins appeared already 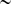
3.4 Gya. However, their frequency and optimization of folding speed continue to increase until 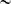
1.5 Gya.

### Protein length and evolution of foldability

The length of the amino acid chain has been reported to influence the folding kinetics of a protein, with longer chains folding more slowly [23], [27]–[29]. We therefore ask if the decrease in SMCO we observed from 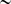
3.8 to 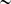
1.5 Gya can be explained by a decrease in the chain length of proteins. Figure 2b shows how domain size has varied in evolution. Folding time measured by SMCO and domain size follow a very similar bimodal trend, with a clear decrease occuring prior to 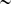
1.5 Gya and a slight increase after the “big bang”. As expected, we find domain size, which equals 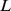 in Equation 1, and SMCO to be correlated with folding rate in agreement with other studies [8], [23] (Figure S6). In line with this correlation, the downhill folders discussed above and shown in Figure 2a as triangles, have a small domain size of less than 100 residues in common.

**Figure 3:**
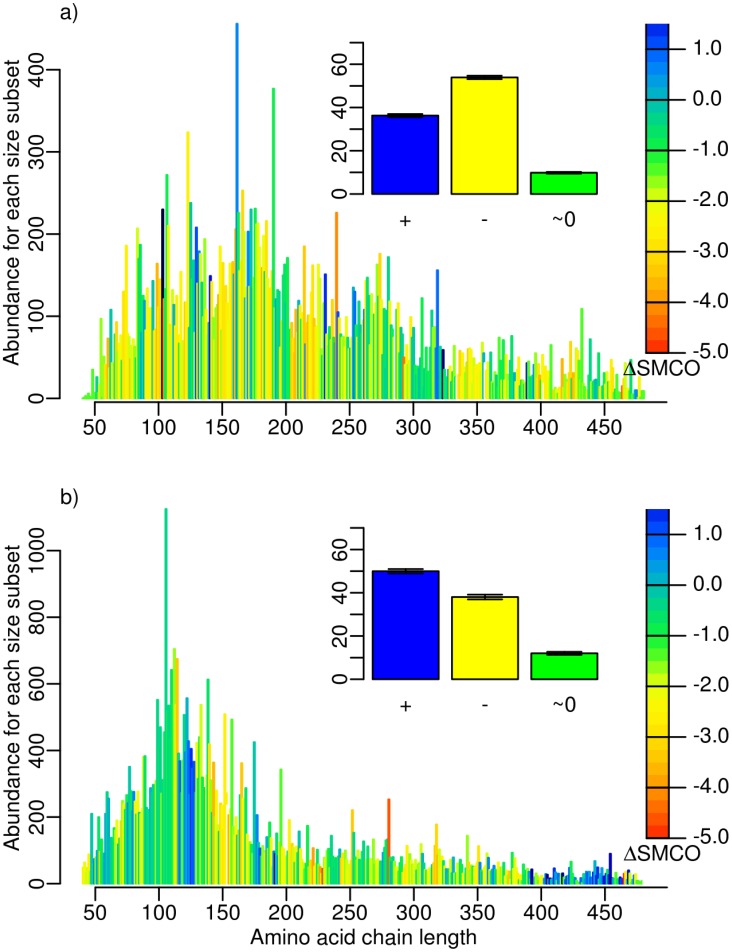
Change in foldability during evolution for subsets of chain size: Distribution of domain length for domains appearing a) 3.8-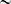
1.5 Gya and b) 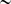
1.5-0 Gya. Abundancies were colored according to the average 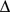
SMCO, the difference between the end points of the polynomial regression of SMCO in this dataset, for the specified initial (a) and later (b) time period. Yellow to red indicates a decrease, and blue an increase in SMCO. The barplots (inset) show the percentage of domains with positive (blue), negative (yellow), and insignificant (green) 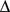
SMCO.

We next eliminated the effect of domain size on the evolutionary trends observed in folding rate to analyze factors other than domain size. To this end, we dissected our dataset according to the amino acid chain length. This analysis was done with all 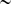
92,000 domains to ensure enough data points for each length. The distributions of chain length are shown in Figure 3a, b for the two time periods before and after the “big bang” (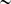
1.5 Gya). The length distribution for proteins appearing before the “big bang” exhibited a peak at around 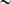
150 amino acids, and shifted later (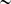
1.5 Gya to the present) to shorter chains with a peak at around 100 aminoacids, underlining the tendency for a decrease of domain size. We note that the resulting average chain length of three-dimensional structures in SCOP, which have been obtained from X-ray or NMR measurements, is smaller than the average length of sequences in genomes [30], apparently due to the increasing experimental difficulties when working with large proteins. We then analyzed evolutionary tendencies for every domain length subset by measuring the variation in the end points of a polynomial regression. The color mapping in Figure 3a indicates an increase (blue), a decrease (yellow-red), or a non-significant change (green) of SMCO. Overall, 85% of the data returned a significant result according to the F-test. During early protein evolution (3.8–1.5 Gya), we found that 54%
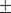
0.3% of all domains in each size subset optimized their foldability during evolution by decreasing their SMCO. Conversely, 37%
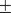
0.4% of domains showed a slow-down in folding, i.e. a significant increase in SMCO. These results confirm the tendencies observed for the full data set (Figure 1a), and hold for different tresholds of identity, namely 95% and 40% (Figures S7, S8). As expected, due to the smaller data set, partitioning domains defined at F and SF levels according to size yielded results that were statistically not significant. In summary, even after dissecting the effect of chain length on changes in SMCO, the tendency of proteins to fold faster during evolution is confirmed.

After the “big bang”, the SMCO and thus foldability showed a overall increase in evolution (Figure 3b), in agreement with results from the total set (Figure 2a). Apparently, fast folding did not represent a major evolutionary constraint during this period. Instead, other constraints must have been optimized at the expense of foldability. We next discuss secondary structure as one factor influencing the impact of foldability on protein structure evolution.

### Secondary structure and evolution of foldability

**Figure 4:**
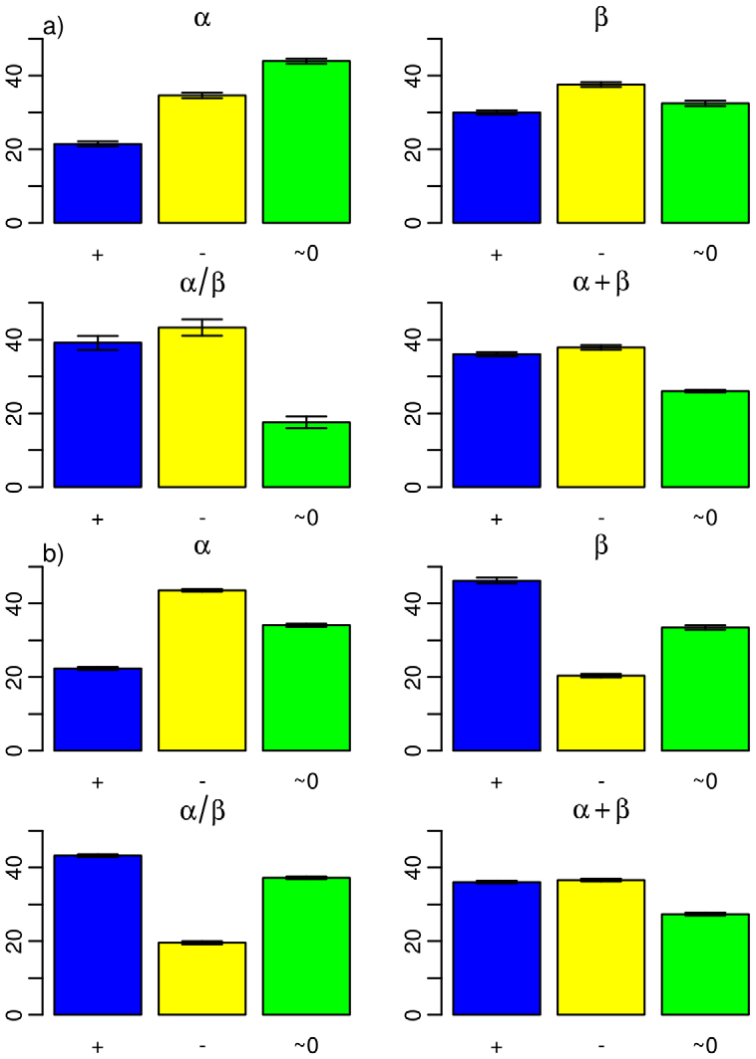
Percentage of all domains with a positive (blue), negative (yellow), and insignificant (green) 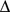
SMCO. a) for 3.8-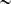
1.5 Gya, and b) 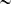
1.5-0 Gya. Each barplot considers one of the four fold classes according to their secondary structure: all-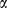, all-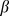, 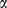/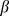, and 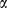
+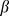, as indicated. The barplots were obtained from domain length distributions analogous to those shown in Figure S3.

Secondary structure composition is another factor reported to have an influence on folding kinetics [23], [27], [28]. We repeated the analysis of domains partitioned by size that was described above for domains in each secondary structure class of SCOP (all-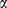, all-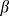, 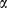/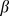, and 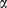
+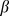 domains) and thereby revealed differences in the evolution of foldability. As shown in Figure 4a, the tendency of a decreasing SMCO before the “big bang” is reproduced for all classes. This result was confirmed at the level 95% identity and 40% identity (Figures S9, S10), though with a significant decrease only for the 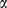
+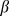 and 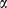 classes at the 40% identity level, i.e. for a much smaller data set. Again, our analysis strongly supports an evolutionary constraint for fast folding of proteins appearing early in evolution, 3.8–1.5 Gya.

Interestingly, we here observe a specialization of protein classes, with all-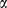 proteins tending to fold faster and all-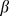 proteins tending to fold more slowly, all of which was supported at the 95% domain level (Figure 4b). Why should the all-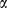 class be under a stronger fast folding constraint than the all-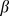 class? Figure S12 shows the average SMCO for each secondary structure class. The all-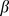 and all-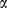 class show the highest and lowest SMCO, respectively, suggesting that all-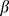 proteins in general fold slower than all-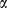 proteins. This is in line with previous findings that containing all-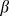 proteins fold more slowly than all-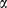 proteins due to long range interactions between all-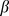 strands that increase contact order [23], [27], [28].

## Discussion

Protein aggregation damages cellular components and can lead to a variety of neuronal diseases [31]–[33]. A way of reducing aggregation is to enhance the kinetic and thermodynamic accessibility of the native fold of a protein. Incremental increases in kinetic or thermodynamic stability of a protein might therefore represent an evolutionary trace reflecting optimization of protein foldability [34].

Here, we confirm the hypothesis that foldability exerts a constraint in the evolution of protein domain structures, as we find a tendency of proteins to on average fold faster than their structural ancestors. As expected, shortening of protein chain length during evolution is an important factor leading to faster folding. However, the exclusion of this protein-size effect preserved the trend of decreasing folding times. Thus, faster folding is not a side effect of chain shortening, but likely acts as an evolutionary constraint in itself. An alternative reason for the decrease of folding times in evolution is the need of proteins for flexibility in order to optimize their function such as enzymatic catalysis or allosteric regulation [35]. Folding speed and flexibility are known to correlate, as the formation of the compact state with no or only minor native contacts is much quicker than the arrangement of the native – often long-range – contacts [36]. Fewer native contacts in turn result in lower stability and may increase conformational flexibility as required for some biological functions [37]. Our analysis of protein folding speed on an evolutionary time line can be similarly carried out for measures of flexibility to test this scenario.

Evolutionary constraints on folding are apparently not uniformly imposed onto the full repertoire of protein structures and during the entire protein history. Instead, our analysis revealed a bimodal evolutionary pattern, with folding speed increasing and decreasing before and after 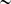
1.5 Gya, respectively. The speed-up of folding was most pronounced for all-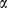 folds. The evolutionary inflexion point coincides with the previously identified protein “big bang”, which features a sudden increase in the number of domain architectures and rearrangements in multi-domain proteins triggered by increased rates of domain fusion and fission. We speculate that the slow down of folding that ensues could be due to cooperative interactions during folding of domains in the emerging multi-domain proteins [38]. Alternatively, the observed slow-down after the “big bang” could be related to the appearance of protein architectures that are known to help proteins to fold, such as chaperones [39], [40] Moreover, protein architectures specific to eukaryotes appeared at 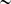
1.5 Gya [16]. The Eukaryotic domain of life has the most elaborate protein synthesis and housekeeping machinery, including enzymes for post-translational modification. This machinery might have mitigated the constraints for fast folding, thereby increasing evolutionary rates of change [34], while preventing misfolding and aggregation prior to attaining the native fold [41].

Finally, we revealed striking evolutionary diversity in protein folding when comparing all-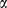 and all-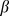 fold classes from 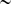
1.5 Gya. Their average folding times diverged after the “big bang”, with the all-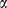 class further decreasing and the all-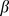 class instead increasing their folding times. This result can support the idea of an optimization of folding that increased the difference in folding time between all-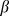 and all-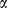 through evolution. As previously shown [22], all-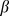 folds have on average higher SMCO and fold slower than their all-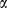-counterparts. This simply results from their different topology and is also the result of our analysis (Figure S12). We here show that earlier in evolution, however, folding times have been more similar and only diverged from each other as late as after 1.8 Gya. But why would all-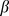 folds have been relieved from the evolutionary constraint of fast folding? Since the “big bang” is responsible for the discovery and optimization of many new functions, including an elaborate protein synthesis and folding machinery, we speculate that the divergence of averge folding times of all-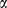 and all-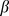 folds probably reflects an optimization of function. This optimization happens to be on the expense of foldability for only the all-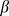 class, the reasons of which remain unknown. One possible scenario would be that all-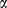 have the tendency to carry out functions that require high flexibility, a property that correlates with few long-range contacts, i.e. high foldability.

An important experimental study by Baker and colleagues [42] tested the idea that rapid folding of biological sequences to their native states does not require extensive evolutionary optimization. Using a phage display selection strategy, the barrel fold of the SH3 domain protein was reproduced with a reduced alphabet of only five amino-acids without any loss in folding rate. Despite extensive changes to protein sequence, experimental manipulation preserved contact order. While these results should not be generalized to the thousands of other fold topologies that exist in nature, they are revealing. They suggest that stabilizing interactions and sequence complexity can be sufficiently small and still enable evolutionary folding optimization. In other words, optimal folding structures can find their way through the free energy landscape without extensive explorations of sequence space. This property of robustness could be a recent evolutionary development, since the SH3 domain F appears very late in our timeline of protein history. Alternatively, it could represent a general structural property. The fact that we now see clear and consistent foldability patterns along the entire timeline supports the existence of limits to evolutionary optimization of folding that are being actively overcome in protein evolution. We conjecture that these limits were initially imposed by the topologies of the early folds, and that structural rearrangements (resulting from insertions, tandem duplication, circular permutations, etc [43]–[46]) offered later on opportunities for fast and robust folding as evolving structures negotiated trade-offs between function and stability.

We end by noting that we cannot exclude overlooking effects on folding times from cooperative folding. These could influence trends of folding times. The SMCO is known to show high correlations with folding times only for single-domain proteins [22]. Developing schemes for estimating folding times from structures comprising more than one domain is a challenge [38] but would enable a more general view onto protein foldability as a constraint throughout evolution. Moreover, our analysis is based on the sequence and structural data that is available. Results might therefore be biased by the choice of proteins and their accessibility. However, the structure of most protein folds and families have been acquired and will not exceed those that are expected [47]. Moreover, our approach allow us to steadily test if the predicted evolutionary trends of foldability are maintained upon inclusion of new sequences and protein folds into the analysis. Interestingly, multiple studies have found folding rates to correlate with stability rather than contact order [48]. Analyzing phylogenomic trends of stability might in this light be an important study to further elucidate evolutionary contraints on protein structure.

## Materials and Methods

### Phylogenomic tree

A most parsimonious phylogenomic tree of F domain structures was reconstructed from a structural genomic census of 3,513 Fs (defined according to SCOP 1.73) in the proteomes of 989 organisms (76 Archaea, 656 Bacteria and 257 Eukarya) with genomes that have been completely sequenced [49]. Similarly, a most parsimonious phylogenomic tree of SF structures (860,497 steps; CI = 0.0255, HI = 0.9745, RI = 0.780, RC = 0.020; g1 = −0.109) was derived from a structural genomic census of 1,915 SFs (defined according to SCOP 1.73) in the proteomes of 1,096 organisms (78 Archaea, 719 Bacteria and 299 Eukarya). The structural census scanned genomic sequences against a library of hidden Markov Models (HMMs) in SUPERFAMILY [50] with probability cutoffs E of 10-4, as described in detail in previous studies [15], [16]. Data matrices of domain abundances were normalized to genome size, coded as multistage phylogenetic characters with characters states ranging from 0 to 29, and used to build rooted trees using maximum parsimony (MP) as optimality criterion in PAUP* [51]. A combined parsimony ratchet and iterative search approach avoided traps in suboptimal regions of tree space. Finally, the age of each domain (nd) was derived directly from its relative position as taxa in reconstructed trees. A PERL script counted the number of nodes from the most ancient domain at the base of the tree to each leaf, providing it in a relative 0-to-1 scale. These relative ages (in nd units) were transformed to geological ages (in Gya) by using molecular clocks of SFs and Fs derived previously [17] and used to construct an evolutionary timeline of domain appearance. A general finding is a sudden explosion of diversity in protein architectures at 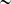
1.5 Gya [19].

### Survey of Size Modified Contact Order

As a measure for the folding time of each protein architecture, we evaluated the size modified contact order (SMCO) of domains indexed in the SCOP database. We used the ASTRAL repositories to download the 92,470 three-dimensional structures classified in SCOP 1.73. The phylogenomic tree was built at the F level on the basis of the same protein structures, i.e. the 1.73 SCOP version. We note that the SMCO calculations are based on single protein domains from SCOP, while many proteins consist of multiple domains. Some studies showed that interactions between domains might affect folding [52]. To test if the evolutionary trends also hold for the subset of domains excluding those which have been structurally solved in multi-domain proteins, we carried out the following steps. We first downloaded the CathDomainList from the website of CATH (http://www.cathdb.info/download), and removed the PDB chains with two or more CATH domains or NMR structures or obsolete PDB entries. We then eliminated redundancy using the PISCES webserver (http://dunbrack.fccc.edu/PISCES.php) [53] using the following cut-offs: Sequence percentage identity: < = 25%, resolution: 0.0 3.0, R-factor: 0.3, sequence length: 40 10,000, Non X-ray entries: excluded, C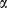-only entries: excluded, cull PDB by chain. We detected SCOP families using HMMs on the PDB chains and removed chains with long non-domain segments, i.e. the length of a segments without any domain assignment should be less than 30. Finally, we removed the chains with two or more SCOP families and the chains with two or more CATH entries. Using this dataset, we revealed the same tendencies in SMCO (Figure S11) as those of the whole dataset (compare Figure 2).

We calculated the average SMCO for each F and SF, and mapped these averages, 3,513 of them for F, and 1,915 for SF, onto timelines derived from corresponding phylogenomic trees. Average SMCO of each F or SF as a function of node distance showed non-linear dependencies that were therefore analyzed using LOESS (locally weighted polynomial regression) [54], [55] to reveal global trends of foldability during evolution. A second-degree polynomial was fitted to the data at each point of the timeline, with a span of 0.7. LOESS resulted in regression function values for each of the 3,513 F or 1,915 SF data points. The results from LOESS revealed a drastic change in SMCO at 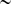
1.5 Gya, a time point of evolution that coincides with the “big bang” of protein domain rearrangements and the rise of Eukarya [19]. We therefore also analyzed our data by two independent linear regressions describing SMCO data points before and after the “big bang”. To validate our results, we repeated the phylogenomic analysis of SMCO using two subsets of protein structures, namely only SCOP domains with 40% of sequence identity (10,570 domains), and those with 95% identity (16,713 domains). In addition, we used one subset of single domain sequences (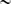
3,500,000 domains) from the TrEMBL [56] database with predicted SMCO [57] the results of which are shown in Figures S3, S4. Only results valid for all four different data sets and thus robust with respect to the selection of protein structures are presented here, if not otherwise noted. For the chain length analysis, we used all 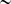
92,000 domains to ensure enough data points for each length. The distributions of chain length are shown in Figure 3a, b. The analysis was repeated 100 times with varying data sample and every dataset (e.g: 95% and 40%). We obtained standard errors of the mean, which are included in Figure 3, 4 and Figures S7, S8, S9, S10.

## Supporting Information

Figure S1 Size Modified Contact Order (SMCO) versus approximate domain age (Gya) a) of domains belonging to the same SF, and b) of domains with less than 95% identity, and c) of domains belonging to the same F. In a) and b), a polynomial regression is shown as black solid line. In c) a linear regression for 3.8 to 1.5 Gya and 1.5 Gya to today was used. The gray area indicates the 95% confidence interval.
(TIF)

Figure S2 Evolutionary changes for an experimental dataset [24] a) Experimental folding rates versus approximate domain age in billion of years ago (Gya). b) Domain size of the same set of 87 proteins versus approximate domain age. A polynomial regression is shown as black line, and the 95% confidence interval as grey shade.
(TIF)

Figure S3 Change in length and foldability during evolution on the SF level using TrEMBL database a) Size Modified Contact Order (SMCO) versus approximative SF domain age in billion of years (Gya). Each data point represents a single domain from the TrEMBL database. b) Average amino-acid chain length for single domains versus SF domain age in Gya. The solid line shows a linear regression, and the dashed line the 95% confidence interval.
(TIF)

Figure S4 Change in length and foldability during evolution on the F level using TrEMBL database a) Size Modified Contact Order (SMCO) versus approximative F domain age in billion of years (Gya). Each data point represents a single domain from the TrEMBL database. b) Average amino-acid chain length for single domains versus F domain age in Gya. The solid line shows a linear regression, and the dashed line the 95% confidence interval.
(TIF)

Figure S5 Tigthness versus approximate domain age (Gya). A polynomial regression is shown as black solid line. The gray area indicates the 95% confidence interval.
(TIF)

Figure S6 Size Modified Contact Order (SMCO) versus folding rate for 87 proteins with experimentally known folding rates [24]. A linear regression is shown as blue dashed line. The solid lines indicates the 95% confidence interval.
(TIF)

Figure S7Distribution of domain length for domains at the 95% similarity appearing a) 3.8-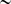
1.5 Gya and b) 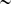
1.5-0 Gya. Abundancies were colored according to the average 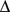
SMCO, the difference between the end points of the polynomial regression of SMCO in this dataset, for the specified initial (a) and later (b) time period. Yellow to red indicates a decrease, and blue an increase in SMCO. The barplot shows the percentages of all domains with positive (blue), negative (yellow), and insignificant (green) 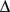
SMCO.(TIF)

Figure S8Distribution of domain length for domains at the 40% similarity appearing a) 3.8-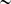
1.5 Gya and b) 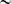
1.5-0 Gya. Abundancies were colored according to the average 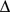
SMCO, the difference between the end points of the polynomial regression of SMCO in this dataset, for the specified initial (a) and later (b) time period. Yellow to red indicates a decrease, and blue an increase in SMCO. The barplot shows the percentages of all domains with positive (blue), negative (yellow), and insignificant (green) 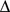
SMCO.(TIF)

Figure S9Percentages of all domains at the 95% similarity with a positive (blue), negative (yellow), and insignificant (green) 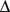
SMCO. a) for 3.8-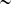
1.5 Gya, and b) 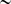
1.5-0 Gya. Each barplot considers one of the four fold classes, all-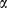, all-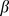, 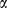/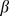, and 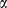
+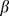, as indicated. See Figure 3 of the main text for how these barplots were obtained.(TIF)

Figure S10Percentages of all domains at the 40% similarity with a positive (blue), negative (yellow), and insignificant (green) 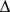
SMCO. a) for 3.8-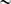
1.5 Gya, and b) 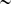
1.5-0 Gya. Each barplot considers one of the four fold classes, 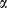, 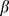, 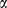/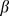, and 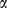
+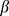, as indicated. See Figure 3 of the main text for how these barplots were obtained.(TIF)

Figure S11 Change in length and foldability during evolution for single domains a) Size Modified Contact Order (SMCO) versus approximate domain age (Gya) for single domains. In a) and b), a polynomial regression is shown as black solid line. The gray area indicates the 95% confidence interval. In comparaison to the data shown in Figure 2 of the main text, domains crystallized within a multi-domain protein have been left out of the analysis.
(TIF)

Figure S12Average SMCO for the four fold classes according to their secondary structure: all-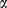, all-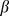, 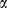/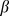 and 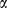
+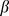. all-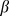 proteins fold significantly more slowly than all-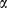 proteins. The Wilcoxon rank-sum test return a p-value
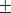
2.2e-16 for every pair of datasets. The higher average SMCO for all-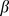 as compared to all-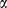 proteins confirms earlier findings [22].(TIF)
