# Supplementary figures and images for: Evolutionary Optimization of Protein Folding
Source: PLoS Comput Biol. 2013 Jan 17;9(1):e1002861. doi: 10.1371/journal.pcbi.1002861 (PMC3547816; doi:10.1371/journal.pcbi.1002861)

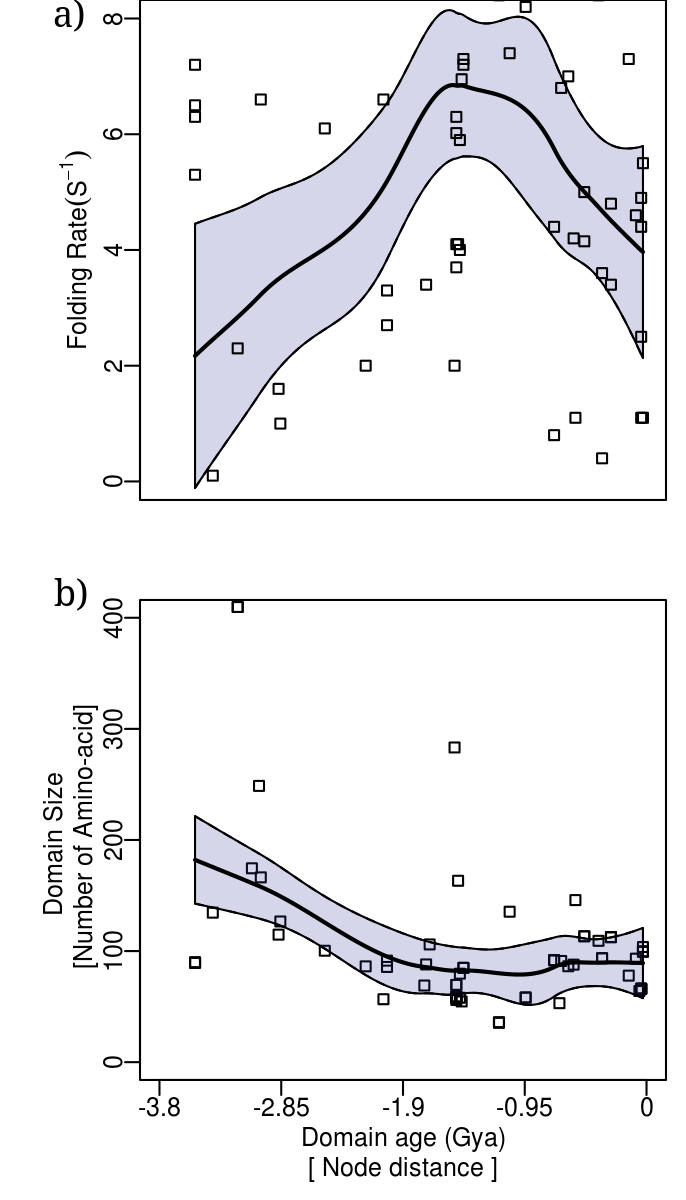

Supplement: Figure S2 — Evolutionary changes for an experimental dataset [24] a) Experimental folding rates versus approximate domain age in billion of years ago (Gya). b) Domain size of the same set of 87 proteins versus approximate domain age. A polynomial regression is shown as black line, and the 95% confidence interval as grey shade. (TIF) [file pcbi.1002861.s002.tif]

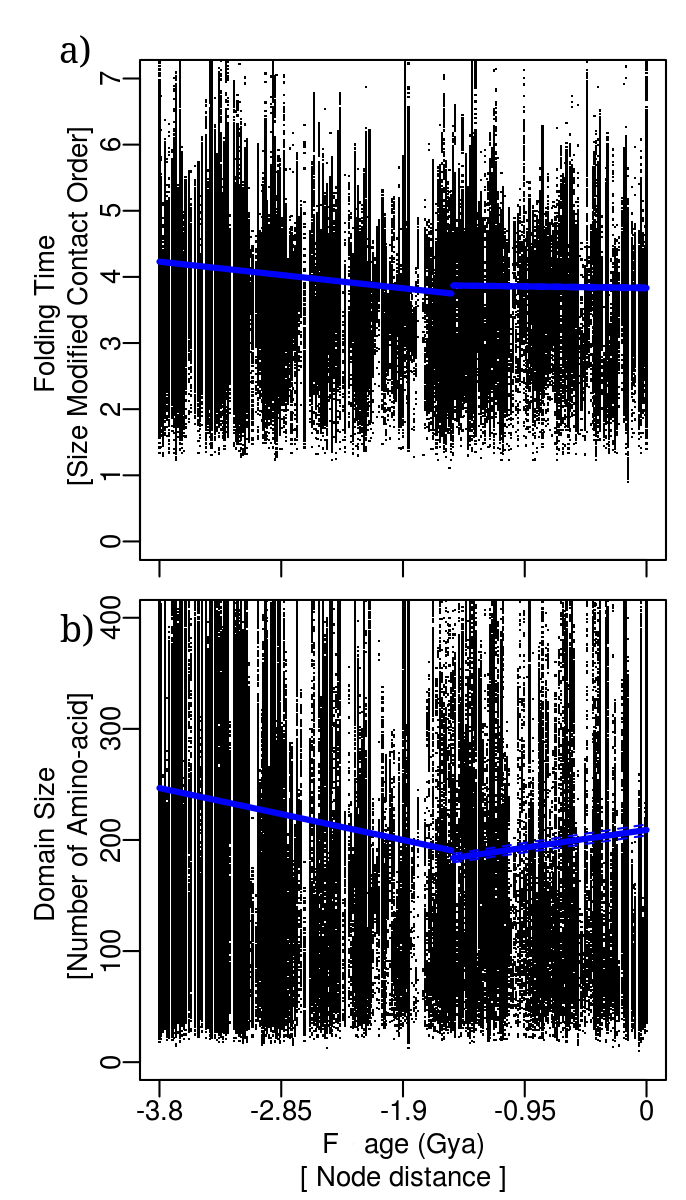

Supplement: Figure S3 — Change in length and foldability during evolution on the SF level using TrEMBL database a) Size Modified Contact Order (SMCO) versus approximative SF domain age in billion of years (Gya). Each data point represents a single domain from the TrEMBL database. b) Average amino-acid chain length for single domains versus SF domain age in Gya. The solid line shows a linear regression, and the dashed line the 95% confidence interval. (TIF) [file pcbi.1002861.s003.tif]

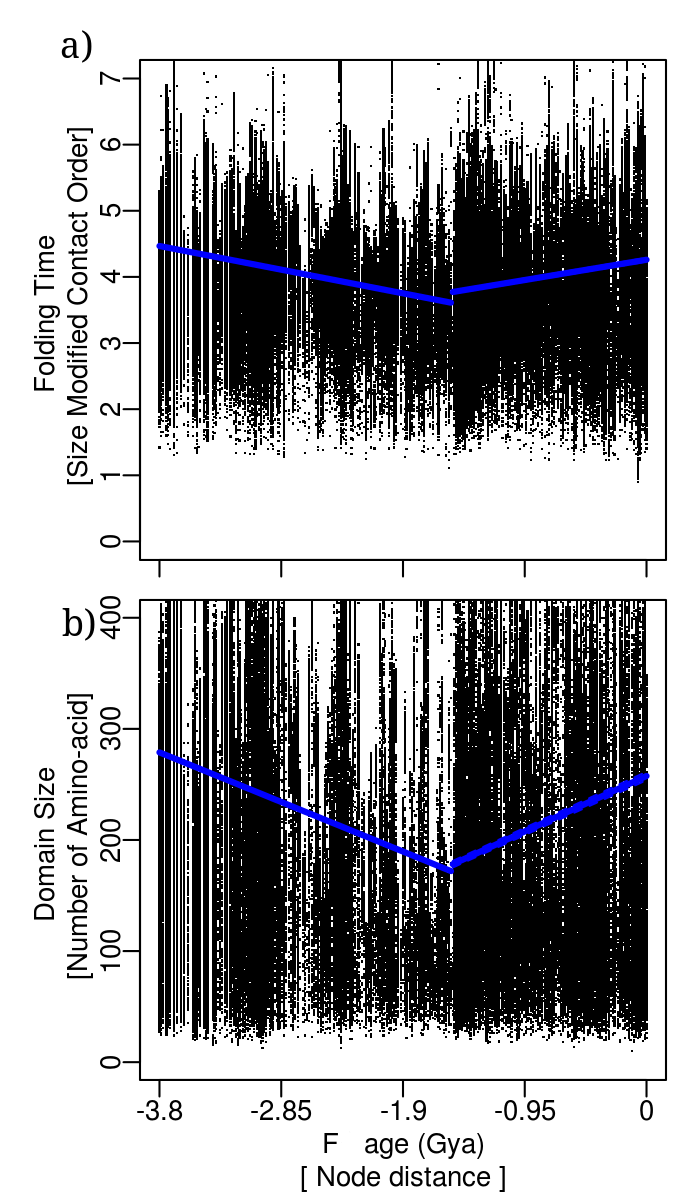

Supplement: Figure S4 — Change in length and foldability during evolution on the F level using TrEMBL database a) Size Modified Contact Order (SMCO) versus approximative F domain age in billion of years (Gya). Each data point represents a single domain from the TrEMBL database. b) Average amino-acid chain length for single domains versus F domain age in Gya. The solid line shows a linear regression, and the dashed line the 95% confidence interval. (TIF) [file pcbi.1002861.s004.tif]

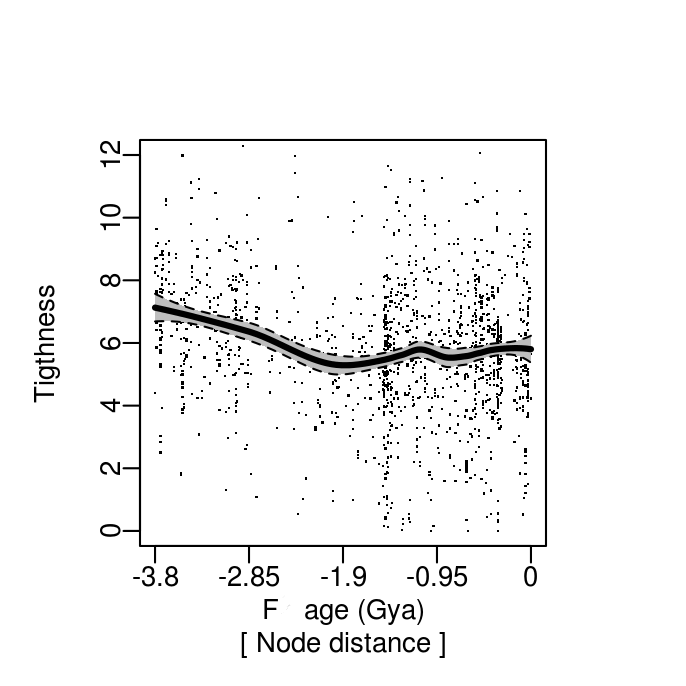

Supplement: Figure S5 — Tigthness versus approximate domain age (Gya). A polynomial regression is shown as black solid line. The gray area indicates the 95% confidence interval. (TIF) [file pcbi.1002861.s005.tif]

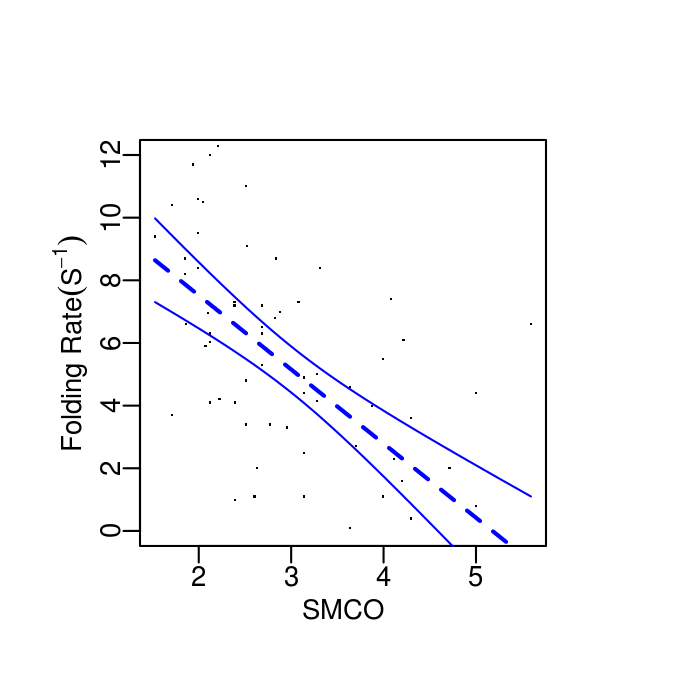

Supplement: Figure S6 — Size Modified Contact Order (SMCO) versus folding rate for 87 proteins with experimentally known folding rates [24]. A linear regression is shown as blue dashed line. The solid lines indicates the 95% confidence interval. (TIF) [file pcbi.1002861.s006.tif]

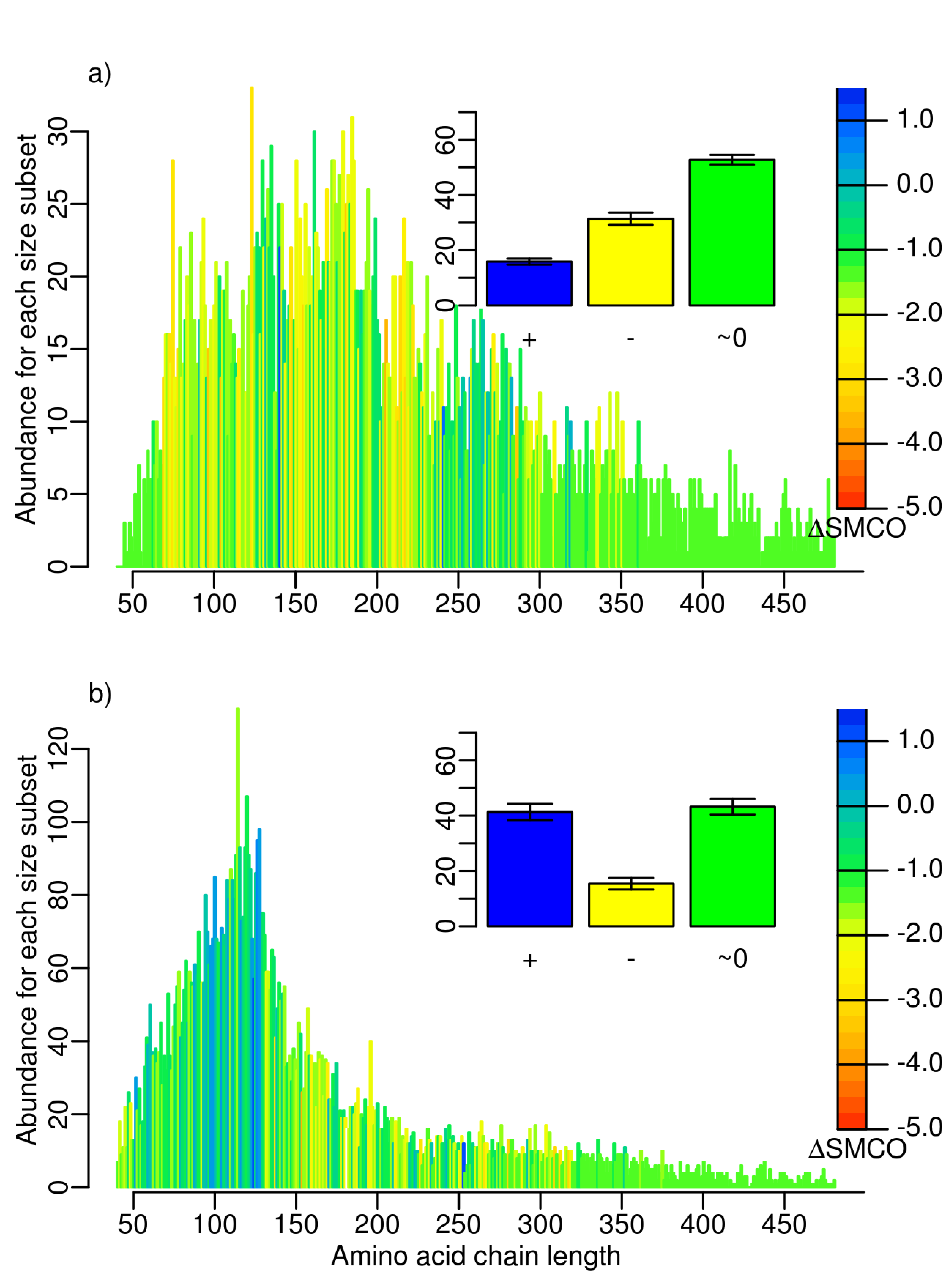

Supplement: Figure S7 — Distribution of domain length for domains at the 95% similarity appearing a) 3.8-1.5 Gya and b) 1.5-0 Gya. Abundancies were colored according to the average SMCO, the difference between the end points of the polynomial regression of SMCO in this dataset, for the specified initial (a) and later (b) time period. Yellow to red indicates a decrease, and blue an increase in SMCO. The barplot shows the percentages of all domains with positive (blue), negative (yellow), and insignificant (green) SMCO. (TIF) [file pcbi.1002861.s007.tif]

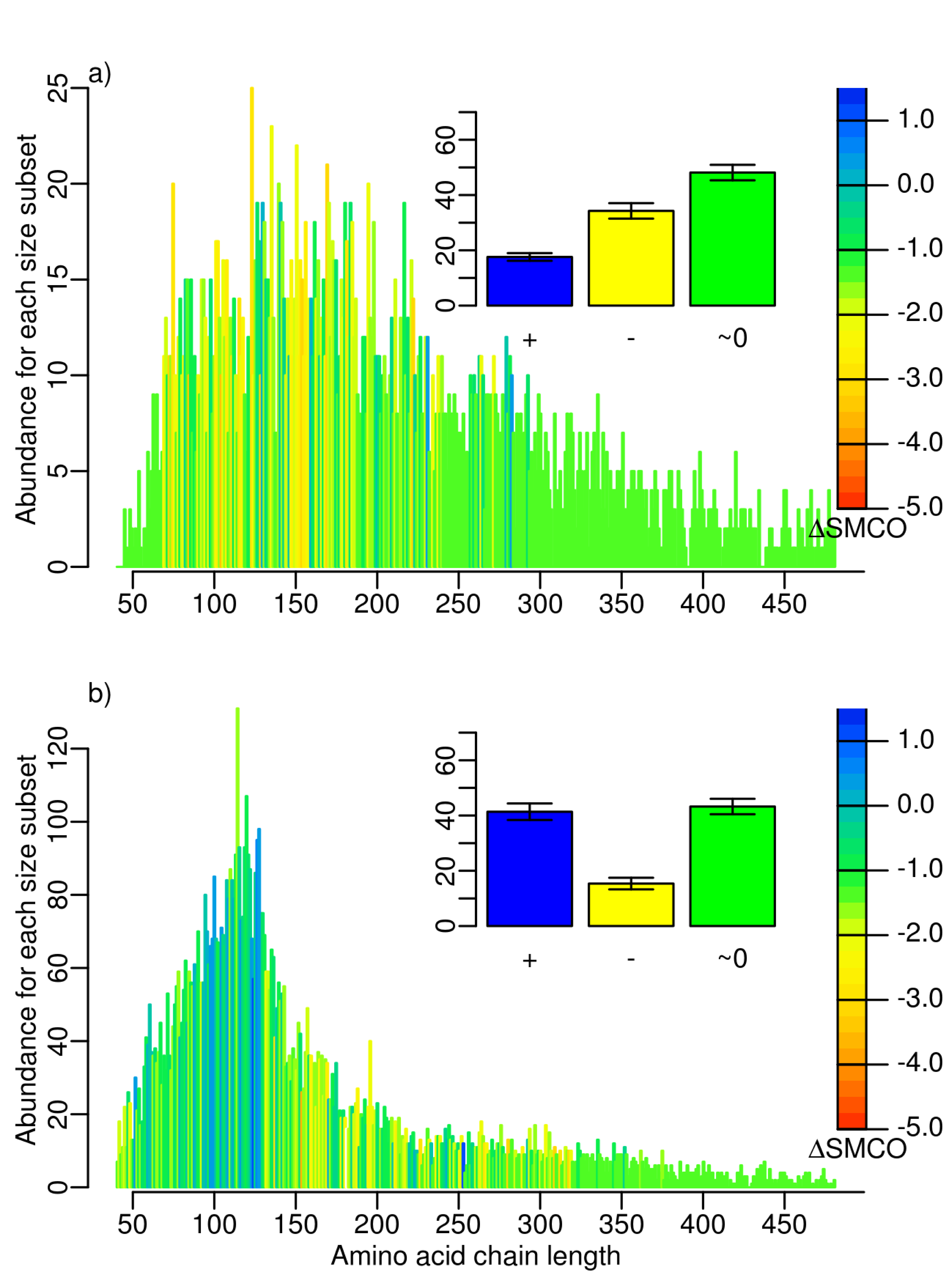

Supplement: Figure S8 — Distribution of domain length for domains at the 40% similarity appearing a) 3.8-1.5 Gya and b) 1.5-0 Gya. Abundancies were colored according to the average SMCO, the difference between the end points of the polynomial regression of SMCO in this dataset, for the specified initial (a) and later (b) time period. Yellow to red indicates a decrease, and blue an increase in SMCO. The barplot shows the percentages of all domains with positive (blue), negative (yellow), and insignificant (green) SMCO. (TIF) [file pcbi.1002861.s008.tif]

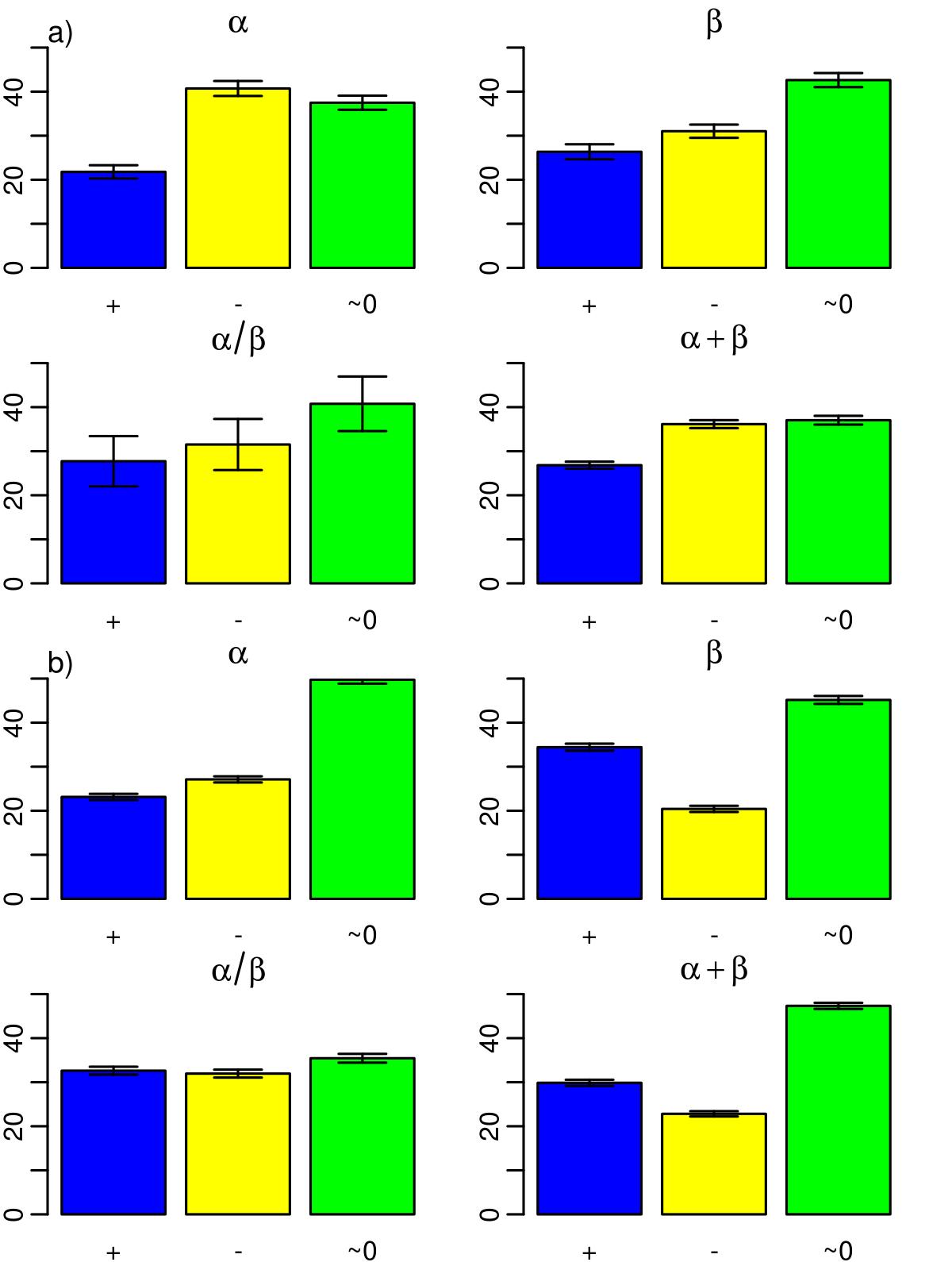

Supplement: Figure S9 — Percentages of all domains at the 95% similarity with a positive (blue), negative (yellow), and insignificant (green) SMCO. a) for 3.8-1.5 Gya, and b) 1.5-0 Gya. Each barplot considers one of the four fold classes, all-, all-, /, and +, as indicated. See Figure 3 of the main text for how these barplots were obtained. (TIF) [file pcbi.1002861.s009.tif]

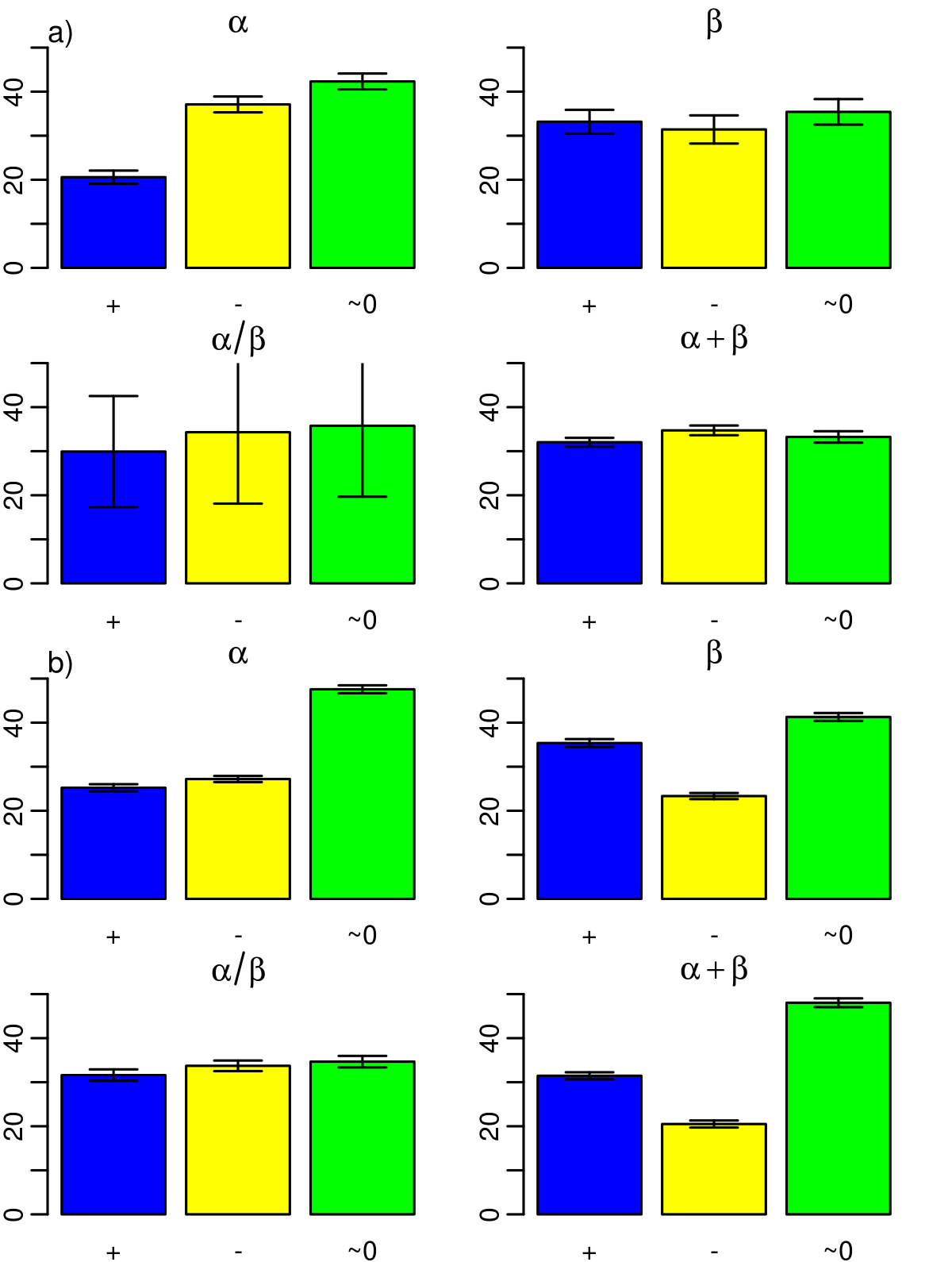

Supplement: Figure S10 — Percentages of all domains at the 40% similarity with a positive (blue), negative (yellow), and insignificant (green) SMCO. a) for 3.8-1.5 Gya, and b) 1.5-0 Gya. Each barplot considers one of the four fold classes, , , /, and +, as indicated. See Figure 3 of the main text for how these barplots were obtained. (TIF) [file pcbi.1002861.s010.tif]

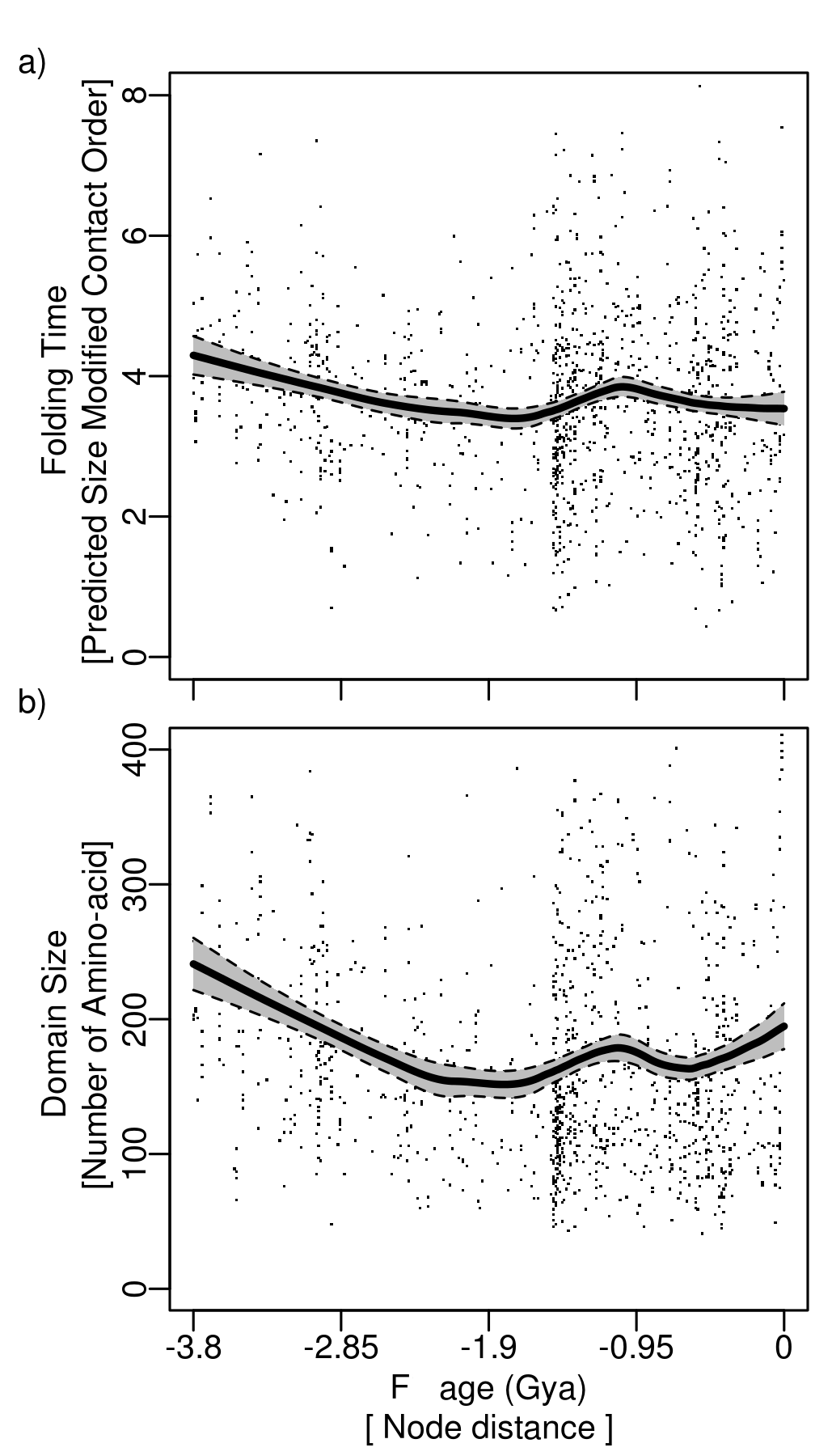

Supplement: Figure S11 — Change in length and foldability during evolution for single domains a) Size Modified Contact Order (SMCO) versus approximate domain age (Gya) for single domains. In a) and b), a polynomial regression is shown as black solid line. The gray area indicates the 95% confidence interval. In comparaison to the data shown in Figure 2 of the main text, domains crystallized within a multi-domain protein have been left out of the analysis. (TIF) [file pcbi.1002861.s011.tif]

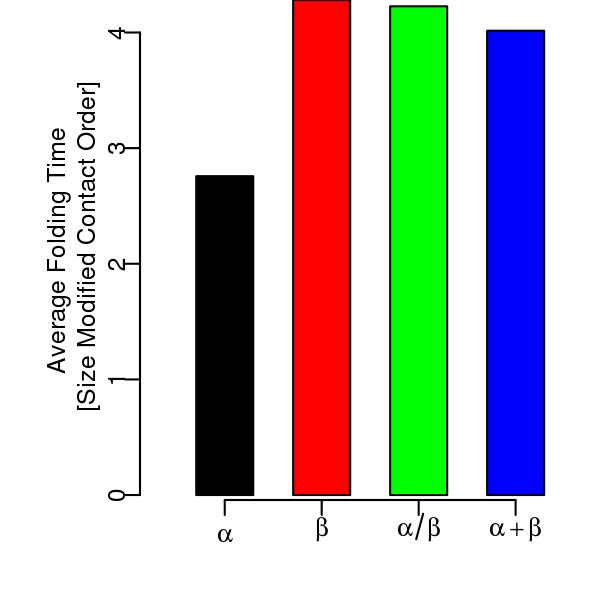

Supplement: Figure S12 — Average SMCO for the four fold classes according to their secondary structure: all-, all-, / and +. all- proteins fold significantly more slowly than all- proteins. The Wilcoxon rank-sum test return a p-value2.2e-16 for every pair of datasets. The higher average SMCO for all- as compared to all- proteins confirms earlier findings [22]. (TIF) [file pcbi.1002861.s012.tif]
